# Medication only improves limb movements while deep brain stimulation improves eye and limb movements during visually-guided reaching in Parkinson’s disease

**DOI:** 10.3389/fnhum.2023.1224611

**Published:** 2023-10-02

**Authors:** Miranda J. Munoz, Rishabh Arora, Yessenia M. Rivera, Quentin H. Drane, Gian D. Pal, Leo Verhagen Metman, Sepehr B. Sani, Joshua M. Rosenow, Lisa C. Goelz, Daniel M. Corcos, Fabian J. David

**Affiliations:** ^1^Department of Physical Therapy and Human Movement Sciences, Northwestern University, Chicago, IL, United States; ^2^USF Health Morsani College of Medicine, University of South Florida, Tampa, FL, United States; ^3^Department of Neurology, Rutgers-Robert Wood Johnson Medical School, New Brunswick, NJ, United States; ^4^Department of Neurological Sciences, Section of Parkinson Disease and Movement Disorders, Rush University Medical Center, Chicago, IL, United States; ^5^Department of Neurology, Northwestern University Feinberg School of Medicine, Chicago, IL, United States; ^6^Department of Neurosurgery, Rush University Medical Center, Chicago, IL, United States; ^7^Department of Neurological Surgery, Northwestern University Feinberg School of Medicine, Chicago, IL, United States; ^8^Department of Kinesiology and Nutrition, University of Illinois at Chicago, Chicago, IL, United States

**Keywords:** Parkinson’s disease, antiparkinson medication, levodopa, subthalamic nucleus deep brain stimulation, saccade, reaching

## Abstract

**Background:**

Antiparkinson medication and subthalamic nucleus deep brain stimulation (STN-DBS), two common treatments of Parkinson’s disease (PD), effectively improve skeletomotor movements. However, evidence suggests that these treatments may have differential effects on eye and limb movements, although both movement types are controlled through the parallel basal ganglia loops.

**Objective:**

Using a task that requires both eye and upper limb movements, we aimed to determine the effects of medication and STN-DBS on eye and upper limb movement performance.

**Methods:**

Participants performed a visually-guided reaching task. We collected eye and upper limb movement data from participants with PD who were tested both OFF and ON medication (*n* = 34) or both OFF and ON bilateral STN-DBS while OFF medication (*n* = 11). We also collected data from older adult healthy controls (*n* = 14).

**Results:**

We found that medication increased saccade latency, while having no effect on reach reaction time (RT). Medication significantly decreased saccade peak velocity, while increasing reach peak velocity. We also found that bilateral STN-DBS significantly decreased saccade latency while having no effect on reach RT, and increased saccade and reach peak velocity. Finally, we found that there was a positive relationship between saccade latency and reach RT, which was unaffected by either treatment.

**Conclusion:**

These findings show that medication worsens saccade performance and benefits reaching performance, while STN-DBS benefits both saccade and reaching performance. We explore what the differential beneficial and detrimental effects on eye and limb movements suggest about the potential physiological changes occurring due to treatment.

## 1. Introduction

Two common treatments for Parkinson’s disease (PD) are antiparkinson medication and subthalamic nucleus deep brain stimulation (STN-DBS). While both treatments effectively improve the motor signs of PD, the mechanisms by which treatment improves behavior may be different. An indirect way to assess these mechanisms is to determine the treatment effects on different effectors, such as the eyes and upper limbs. This will help elucidate how treatments are affecting multiple neural circuits. However, treatment effects on eye and upper limb performance have typically been assessed in separate experiments with different tasks. We aimed to assess and better understand how the different treatments of PD affect both eye and upper limb movements during the same movement task using comparable outcomes. We focused on two aspects of eye and upper limb movement: latency/reaction time (RT) and peak velocity.

The results from separate eye only and upper limb only movement tasks suggest that antiparkinson medication may have differential effects on the eyes and upper limbs. We have recently shown that medication increased saccade latency (Munoz et al., 2022), confirming the findings of previous studies (Müller et al., 1994; Michell et al., 2006; Hood et al., 2007; Dec-Ćwiek et al., 2017; Lu et al., 2019; Waldthaler et al., 2019). However, other studies have reported that medication does not have a significant effect on saccade latency (Gibson et al., 1987; Rascol et al., 1989; Temel et al., 2009; van Stockum et al., 2012; Cubizolle et al., 2014; Bakhtiari et al., 2020), which could be due to suboptimal medication doses (Munoz et al., 2022). Conversely, the medication effect on simple RT tasks evaluating upper limb movement has typically lacked statistical significance, however, there has been a consistent pattern of medication decreasing simple RT within all (Velasco and Velasco, 1973; Bloxham et al., 1987; Pullman et al., 1988, 1990; Starkstein et al., 1989; Jahanshahi et al., 1992; Fern-Pollak et al., 2004; Ingram et al., 2021) but one study (Müller and Harati, 2020). Additionally, one study found this decrease in latency with medication to be statistically significant (Montgomery and Nuessen, 1990). Overall, these findings suggest that medication significantly increases saccade latency and decreases upper limb simple RT, but not significantly.

During the visually-guided saccade task, medication typically decreased saccade peak velocity, either significantly (Munoz et al., 2022) or not significantly (Dec-Ćwiek et al., 2017; Lu et al., 2019). Conversely, studies reported that medication typically increased the peak velocity of simple upper limb movements during reach-to-grasp tasks (Castiello et al., 2000; Negrotti et al., 2005; Schettino et al., 2006), arm abduction to match a target (Baroni et al., 1984), wrist flexion (Berardelli et al., 1986; Johnson et al., 1994), elbow flexion (Robichaud et al., 2002; Vaillancourt et al., 2004), and reach- or point-to-target tasks (Kelly et al., 2002; Camarda et al., 2005). Similarly, medication has improved finger tapping speed and pronation-supination speed (Brzezicki et al., 2023). Taken together, previous literature suggests that medication will decrease saccade peak velocity but increase upper limb peak velocity. However, the effects of medication on both eye and limb movements have not been tested using a single task and cohort of participants with PD.

Unlike studies examining the effects of medication, the previous literature evaluating the effects of STN-DBS suggest that bilateral STN-DBS may have similar effects on eye and upper limb movements. Using the visually-guided saccade task, most studies have shown that STN-DBS decreased saccade latency compared to OFF stimulation, typically significantly (Fawcett et al., 2007, 2010; Sauleau et al., 2008; Temel et al., 2008, 2009; Yugeta et al., 2010; Antoniades et al., 2012a,b, 2015; Dec-Ćwiek et al., 2017; Goelz et al., 2017; Bakhtiari et al., 2020), but occasionally not significantly (Rivaud-Péchoux et al., 2000; Lohnes and Earhart, 2012; Pinkhardt et al., 2012; Nilsson et al., 2013). Similarly to saccade latency, studies found that bilateral STN-DBS decreased upper limb RT during a simple RT task, either significantly (Brown et al., 1999; Temel et al., 2006; Antoniades et al., 2012b) or trending toward significance (Kumru et al., 2004). Additionally, one study compared the effect of STN-DBS on visually-guided saccade latency and button press RT. They found that STN-DBS decreased both latency and RT (Antoniades et al., 2012b), but other aspects of movement, such as peak velocity, were not evaluated. Overall, these findings suggest that bilateral STN-DBS will decrease both saccade latency and reach RT.

Previous studies using the visually-guided saccade task reported that bilateral STN-DBS increased saccade peak velocity, either significantly (Nilsson et al., 2013) or not significantly (Pinkhardt et al., 2012; Dec-Ćwiek et al., 2017). Similarly, STN-DBS has repeatedly been found to increase peak velocity of upper limb movements, such as reach-to-grasp (Dafotakis et al., 2008), finger tapping (Dafotakis et al., 2008; Tamás et al., 2016), repetitive pointing (Dafotakis et al., 2008), hand grasping (Tamás et al., 2016), elbow flexion (Vaillancourt et al., 2004), pronation/supination (Tamás et al., 2016), and cued upper limb joint movements (Joundi et al., 2012). This would be expected as STN-DBS is a common treatment of PD because it has been proven to improve motor function (Limousin et al., 1995; Pollak et al., 1996; Krack et al., 1998; Brown et al., 1999; Thobois et al., 2002; Rodriguez-Oroz et al., 2005). Taken together, previous literature suggests that STN-DBS will increase saccade and upper limb peak velocity. However, the effects of STN-DBS on eye and upper limb movements have mostly been evaluated separately, limiting direct comparisons.

This study investigated the effects of antiparkinson medication and bilateral STN-DBS on the oculomotor and skeletomotor systems during a task requiring both eye and upper limb movement. First, we determined the effect of medication on saccade and reach latency/RT and peak velocity. We hypothesized that medication would increase saccade latency while having no effect on or decreasing reach RT and would decrease saccade peak velocity while increasing reach peak velocity. Second, we determined the effect of bilateral STN-DBS on saccade and reach latency/RT and peak velocity. We hypothesized that STN-DBS would decrease both saccade latency and reach RT and would increase both saccade and reach peak velocity. Third, we determined the relationship between saccade latency and reach RT for a cohort of people with PD tested OFF and ON medication, a cohort of people with PD tested OFF and ON STN-DBS, and healthy controls to evaluate whether treatment has an impact on this relationship.

## 2. Materials and methods

### 2.1. Participants

Northwestern University and Rush University Medical Center Institutional Review Boards approved this study, and all experiments were completed in accord with the Helsinki Declaration of 1975. We obtained informed consent from all participants. Participants with PD were recruited from the movement disorder clinics at both institutions. Participants with PD were examined by a movement disorders neurologist and met the United Kingdom PD Society brain bank clinical diagnostic criteria (Hughes et al., 1992a,b; Berardelli et al., 2013) but had no neurological comorbidities, while healthy controls had no reported history of any neurological disorders. All participants had (1) normal or corrected-to-normal visual acuity, (2) no eye movement abnormalities, such as blepharospasm, double vision, and/or eyelid opening apraxia, and (3) the ability to understand and perform the experimental task during intake. All participants were right-hand dominant, as confirmed by the Edinburgh Handedness Inventory (Oldfield, 1971).

To examine the medication effect, 34 individuals with PD (28 males, 6 females) who were treated with antiparkinson medication completed testing of the visually-guided reaching task (Table 1). To examine the STN-DBS effect, we recruited 14 individuals with PD who had high-frequency bilateral STN-DBS. Three participants were unable to complete testing OFF STN-DBS. Therefore, the STN-DBS effect analysis included 11 individuals with PD (11 males) who were treated with bilateral STN-DBS (Table 1). Individuals were tested 8 months post-surgery on average (range: 6–12 months). Finally, we also tested 17 older adult healthy controls, but 3 were excluded due to a high Movement Disorder Society-Unified Parkinson’s Disease Rating Scale (MDS-UPDRS) Part III motor score (>12), a low Montreal Cognitive Assessment (MoCA) score (<18), or fatigue preventing the participant from successfully completing the task. The final healthy control group included 14 participants (12 males, 2 females) (Table 1).

**TABLE 1:** Characteristics of participants with Parkinson’s disease in the medication and STN-DBS effect analyses and healthy controls.

	Medication effect (*n* = 34)	STN-DBS effect (*n* = 11)	Healthy controls (*n* = 14)
Sex (M/F)	28/6	11/0	12/2
Age (years)	65.88 ± 3.86	66.64 ± 3.17	65.43 ± 4.24
Disease duration (years)	7.12 ± 4.30	10.27 ± 4.84	.
Time since surgery (months)	.	8.27 ± 1.74	.
MoCA	27.68 ± 1.92	27.00 ± 2.00	27.21 ± 1.63
**MDS-UPDRS Part III**			
Off meds	43.24 ± 15.06	.	2.93 ± 2.30
On meds	32.03 ± 11.55	.	.
Off meds, Off DBS	.	50.55 ± 14.27	.
Off meds, On DBS	.	19.55 ± 8.17	.
Levodopa equivalent daily dose (mg)	790.00 ± 658.46	405.45 ± 288.92	.

Values are mean ± standard deviation. DBS, deep brain stimulation; MDS-UPDRS, Movement Disorder Society-Unified Parkinson’s Disease Rating Scale; mg, milligrams; MoCA, Montreal Cognitive Assessment.

### 2.2. Experimental conditions

To determine the medication effect, data collection took place over 3 days: 1 day for intake and 2 days for testing. During intake, participants with PD were consented, were administered the MoCA, and practiced the experimental tasks while ON medication. Testing occurred over the next 2 days: 1 day OFF medication and 1 day ON medication, with the order of medication condition randomized across participants. For OFF medication testing, participants withdrew from all antiparkinson medications for at least 12-h before the start of testing (Langston et al., 1992). For ON medication testing, participants took their medications as usual. To verify that participants were in the “off state” or “on state,” the experimenter confirmed with the participant that they felt “off” or “on” before testing began. MDS-UPDRS Part III was administered right before testing each day.

To determine the bilateral STN-DBS effect, data collection took place over 5 days: 1 day for intake and 4 days for testing. Intake was completed ON medication and ON bilateral STN-DBS and otherwise was identical to the intake day of the medication effect participants. Testing occurred over the next 4 days: 1 day OFF STN-DBS, 1 day with only the left stimulator on, 1 day with only the right stimulator on, and 1 day ON bilateral stimulation with the order of STN-DBS condition randomized across subjects. Only data from the OFF STN-DBS and ON bilateral STN-DBS conditions are presented in this manuscript. Stimulators were turned OFF at least 3-h prior to testing (Temperli et al., 2003). ON bilateral stimulation testing was completed on clinical stimulation settings (Table 2). All testing was completed OFF medication after at least 12-h overnight withdrawal (Langston et al., 1992). MDS-UPDRS Part III was administered right before testing each day.

**TABLE 2:** Clinical stimulation settings for the participants in the STN-DBS effects analysis.

ID	Left stimulator clinical settings					Right stimulator clinical settings				
	Amp (V or mA)	Freq (Hz)	PW (μs)	+ Contact	− Contact	Amp (V or mA)	Freq (Hz)	PW (μs)	+ Contact	− Contact
1	3.0*	130	60	1	0	4.0*	130	60	10	9
2	2.4	130	60	Case	2abc	2.2	130	60	Case	10abc
3	3.0*	125	60	1	2	3.2*	125	60	Case	9
4	2.5*	130	60	0	2, 3	2.9*	130	90	11	9
5	2.9	160	90	Case	3ac	2.0	130	60	Case	10abc
6	2.4	130	60	Case	3abc	3.2	130	90	Case	10abc
7	2.9	130	60	Case	3abc	2.9	130	60	Case	10abc
8	3.0	130	60	Case	2abc	3.1	130	60	Case	10ab
9	2.1	130	60	Case	2c	2.7	130	60	Case	10c
10	3.8	130	60	Case	2c, 4	2.8	130	60	Case	10abc
11	3.6	130	60	Case	2c	2.5	180	60	Case	10abc

The above left and right settings were used for bilateral STN-DBS testing.

* Indicates that the participant had constant voltage stimulation and the amplitude value is in volts. All other participants had constant current stimulation and the amplitude value is in milliamperes. Amp, amplitude; Freq, frequency; Hz, hertz; μs, microsecond; mA, milliamperes; − Contact, negative contact; + Contact, positive contact; PW, pulse width; V, volts. Those with constant current stimulation had segmented electrodes, which are represented by the segment names a, b, and c.

For healthy controls, intake and testing were completed in 1 day. Testing for all participants included a series of 6 different eye and upper limb movement tasks, but only data from one, the visually-guided reaching task, is reported in this manuscript.

### 2.3. Instrumentation

Participants sat upright in a height-adjustable chair with their chin placed on a chin rest to minimize head movement. We recorded binocular eye movements at 500 Hz using an infrared camera-based eye-tracking system (Eyelink II, SR Research Ltd, Ottawa, ON, Canada). We recorded head, upper limb, and robotic arm movements at 240 Hz using a three-dimensional motion capture system (Optotrak 3020, Northern Digital, Waterloo, ON, Canada). To capture head and upper limb movements, participants had infrared light-emitting diodes (iLED) attached to the eye tracking system on the head and another iLED attached to their right index finger adjacent to their fingernail. To capture the robotic arm movements, another iLED was attached to the end of the robotic arm. Eye, head, upper limb, and robotic arm movements were synchronized, after down-sampling the eye-tracking data to 240 Hz, and stored using The MotionMonitor (Innovative Sports Training, Chicago, IL, USA).

The task was presented using 3 mm green LEDs (70 mcd), the first as the central fixation LED mounted on a central fixation stand and the second as the peripheral target LED, which was attached to the tip of a robotic arm (Thermo CRS, Burlington, ON, Canada). The central fixation LED was positioned at eye level, 42 cm away from the chin rest and participant. The task was completed in the dark.

### 2.4. Visually-guided reaching task

Each trial began with the participant fixating their eyes on the central LED and their right index finger resting on the central fixation stand for a time interval between 2000 and 3000 ms, after which the central LED was extinguished. After a 200 ms gap, a peripheral target LED was presented to the right along the horizontal plane. The participant was instructed to “look and touch the target LED as accurately as possible at a comfortable speed.” The target LED was presented for 2000 ms at a 10° or 15° visual angle (7.41 or 11.25 cm) from the central LED at random, but only trials with the 15° target were analyzed in this manuscript for simplicity. We chose a 15° visual angle due to equipment limitations for accurate eye-tracking and we used the 10° visual angle to introduce a choice to prevent memorization of the target location. We chose to analyze only the 15° target trials because these trials were more difficult than the 10° target trials, making them likely more sensitive to differences in behavior. Participants performed one block of 20 trials, 10 trials for each target LED location, to prevent fatigue while having enough trials to reach statistical significance according to pilot testing. Participants were given a short break after 10 trials and the lights were turned on to limit adaptation to the dark. Before the block of 20 trials, all participants completed practice trials until they could confidently perform the task correctly.

### 2.5. Data processing

The eye and upper limb data were processed using a custom MATLAB script (The MathWorks Inc., Natick, MA, USA). Eye and upper limb position data were filtered using a 20 Hz low-pass second-order, zero-phase Butterworth filter. The filtered position data were differentiated to calculate velocity. Tangential velocity was calculated in 2 dimensions for the eye movement data and in 3 dimensions for upper limb movement data.

Using the eye and upper limb position data and the tangential velocity data, an approximate estimate of the eye and upper limb movement onset and offset was marked using visual inspection to create a region of interest in time. We computed eye and upper limb peak velocities within this region of time. From the peak velocities, we identified the first time point when velocity went below 5% of the peak velocity, which was defined as the eye or upper limb movement onset. Saccade latency and reach RT were defined as the time difference between target LED onset and the algorithmically identified movement onset of the saccade or reach. During visual inspection, if it was clear the trial was not performed correctly, it was marked invalid. For instance, if the participant moved their eyes or upper limb prior to the presentation of the target LED or if they did not complete the reach in the allotted time. All trials marked invalid were excluded.

After visual inspection, trials were further excluded based on predetermined criteria: (1) saccade latency was < 90 ms (Munoz et al., 1998) or >1000 ms, (2) reach RT was < 200 ms or >1000 ms, or (3) reach RT occurred >500 ms before saccade latency. Based on visual inspection of the data, trials were excluded as outliers if reach peak velocity was >1 m/s or reach end point error was >5 cm. Our outcome variables were saccade latency, reach RT, saccade peak velocity, and reach peak velocity.

### 2.6. Statistical analysis

Linear mixed-effect regression models were used to assess each of the saccade and reach outcomes. For the medication analysis, the fixed effect was medication condition (OFF and ON medication). For the STN-DBS analysis, the fixed effect was STN-DBS condition (OFF and ON bilateral STN-DBS). The random effect was participant in both analyses. To meet the distributional assumptions for mixed modeling, if the observed data was right skewed, the data was transformed using a log function. This occurred for saccade latency, reach RT, and reach peak velocity. If the observed data was left skewed, the data was transformed using a squared function. This was the case for saccade peak velocity. The statistics presented are in log or squared scales, along with the estimated difference transformed back to the original scale (Est diff_BT_). The relationship between saccade latency and reach RT was assessed across medication conditions, across STN-DBS conditions, and in the healthy controls using mixed-effect regression models. Mixed models were also used to assess the interaction between the treatment and saccade latency effects on reach RT. The significance of the results was identical between the original scale and the log transformation scale, so we present the data and statistics in the original scale for ease of interpretation. All statistical analyses were performed using SAS^®^ (version 9.4, SAS Institute, Cary, NC, USA).

## 3. Results

### 3.1. Medication effect

**FIGURE 1:**
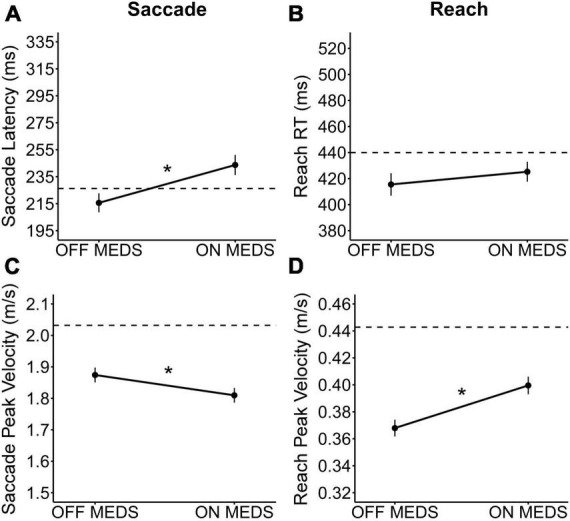
The medication effect on saccades and reaching. The observed medication effect on **(A)** saccade latency, **(B)** reach RT, **(C)** saccade peak velocity, and **(D)** reach peak velocity. The plots show the mean medication effect and standard errors, with observed means from healthy controls presented for reference (dashed line). * Indicates that the medication effect was statistically significant (*p* < 0.05).

Medication significantly increased saccade latency [Est diff_BT_ = 25.98 ms; *F*_(1,542)_ = 17.50; *p* < 0.001; Figure 1A] but had no statistically significant effect on reach RT [Est diff_BT_ = 13.96 ms; *F*_(1,542)_ = 2.96; *p* = 0.086; Figure 1B] compared to OFF medication. Additionally, medication significantly decreased saccade peak velocity [Est diff_BT_ = −0.04 m/s; *F*_(1,542)_ = 4.75; *p* = 0.030; Figure 1C] but significantly increased reach peak velocity [Est diff_BT_ = 0.03 m/s; *F*_(1,542)_ = 23.00; *p* < 0.001; Figure 1D] compared to OFF medication. Observationally, with medication, the mean saccade latency and peak velocity became further from healthy control means, while mean reach peak velocity became closer to healthy control means.

### 3.2. Bilateral STN-DBS effect

**FIGURE 2:**
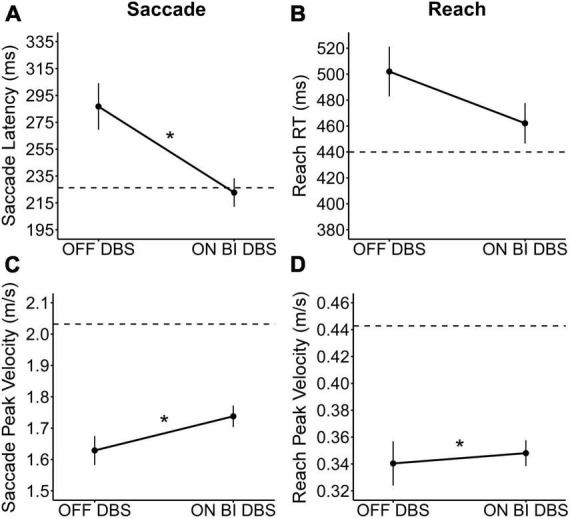
The bilateral STN-DBS effect on saccades and reaching. The observed STN-DBS effect on **(A)** saccade latency, **(B)** reach RT, **(C)** saccade peak velocity, and **(D)** reach peak velocity. The plots show the mean STN-DBS effect and standard errors, with observed means from healthy controls presented for reference (dashed line). Participants were OFF medication during OFF and ON bilateral STN-DBS testing. * Indicates that the bilateral STN-DBS effect was statistically significant (*p* < 0.05).

Bilateral STN-DBS significantly decreased saccade latency [Est diff_BT_ = −38.89 ms; *F*_(1,159)_ = 7.75; *p* = 0.006; Figure 2A] but had no statistically significant effect on reach RT [Est diff_BT_ = −29.93 ms; *F*_(1,159)_ = 2.91; *p* = 0.090; Figure 2B] compared to OFF STN-DBS. Additionally, bilateral STN-DBS significantly increased saccade peak velocity [Est diff_BT_ = 0.09 m/s; *F*_(1,159)_ = 4.20; *p* = 0.042; Figure 2C] and significantly increased reach peak velocity [Est diff_BT_ = 0.03 m/s; *F*_(1,159)_ = 6.48; *p* = 0.012; Figure 2D] compared to OFF STN-DBS. Observationally, all three statistically significant findings resulted in performance changes with STN-DBS that became closer to healthy control performance.

### 3.3. Saccade latency effect on reach RT

**FIGURE 3:**
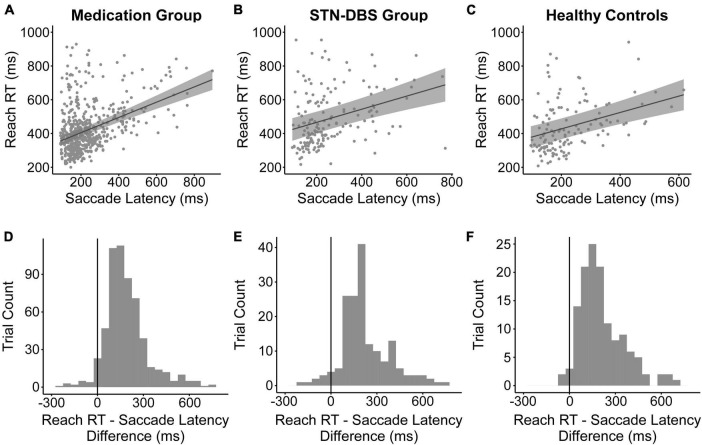
Relationship between saccade latency and reach reaction time. The relationship was significant for the data analyzed for the **(A)** medication group, **(B)** STN-DBS group, and **(C)** healthy controls. The plots depict the model-estimated relationship (solid black line) and 95% confidence interval (gray shaded area). The observed trial-level data are overlaid onto the plots (gray filled circles). The histograms show the difference in time between the reach RT and saccade latency for the **(D)** medication group, **(E)** STN-DBS group, and **(F)** healthy controls. All values over zero show that saccade initiation occurs before reach initiation.

Since the direction of change in mean reach RT followed the change in mean saccade latency for both treatments, we wanted to determine the relationship between saccade latency and reach RT. Saccade latency had a significant positive relationship with reach RT across the OFF and ON data of the participants in the medication analysis [*ß* = 0.452; estimated intraclass correlation coefficient (ICC) = 30.58%; *p* < 0.001; Figure 3A] and of the participants in the STN-DBS analysis [*ß* = 0.389; ICC = 43.26%; *p* < 0.001; Figure 3B]. To determine if this relationship was affected by treatment, we also determined whether there was an interaction between the treatment and saccade latency effects on reach RT. There was no interaction between either medication condition and saccade latency (*p* = 0.171) or STN-DBS condition and saccade latency (*p* = 0.211). Finally, the positive relationship between saccade latency and reach RT was also seen in the healthy controls (*ß* = 0.484; ICC = 65.74%; *p* < 0.001; Figure 3C). In the vast majority of trials, saccade initiation occurred before reach initiation for the participants in the medication analysis (Figure 3D), participants in the STN-DBS analysis (Figure 3E), and healthy controls (Figure 3F).

## 4. Discussion

We investigated the effects of antiparkinson medication and bilateral STN-DBS on visually-guided saccades and reaching movements during a visually-guided reaching task. Notably, this is the first report of both the medication and STN-DBS effects on the eye and upper limb movements during the same task. We have three key findings. First, antiparkinson medication had an adverse effect on saccade performance and a beneficial effect on one aspect of reach performance. Antiparkinson medication increased saccade latency, decreased saccade peak velocity, and increased reach peak velocity. Second, STN-DBS had a beneficial effect on saccade performance and a beneficial effect on one aspect of reach performance. Bilateral STN-DBS decreased saccade latency, increased saccade peak velocity, and increased reach peak velocity. Third, we confirmed that there was a positive relationship between saccade latency and reach RT across treatment groups and healthy controls, in which saccade initiation preceded limb initiation. Importantly, the positive relationship remained unaffected by treatment. Finally, we discuss the possible parallel mechanisms underlying the similar effects of medication and STN-DBS on reach peak velocity and the possible unique mechanisms underlying the differential effects of medication and STN-DBS on saccade performance.

### 4.1. Differential effects of medication on saccade and reach performance

We found that antiparkinson medication adversely impacted saccade performance and benefitted one aspect of reach performance (Figure 1). Our findings confirmed our prediction that antiparkinson medication would increase saccade latency. Our recent study and other previous studies have shown that medication increases saccade latency during a saccade only task (Müller et al., 1994; Michell et al., 2006; Hood et al., 2007; Dec-Ćwiek et al., 2017; Lu et al., 2019; Waldthaler et al., 2019; Munoz et al., 2022). The effect of medication on saccade peak velocity has been less established. Three previous studies have found no statistically significant effect on saccade peak velocity (Rascol et al., 1989; Dec-Ćwiek et al., 2017; Lu et al., 2019), but, observationally, 2 of the 3 showed a slight decrease in peak velocity with medication during the visually-guided saccade task (Dec-Ćwiek et al., 2017; Lu et al., 2019). The third study did not show a slight decrease, possibly because of the small medication dosage given to the participants (Rascol et al., 1989). Our recent study found a statistically significant decrease in peak velocity with medication on the visually-guided saccade task (Munoz et al., 2022). In the present study, we extended previous saccade findings. Medication increases saccade latency and decreases saccade peak velocity not only during a saccade task, but also in a task requiring eye-hand coordination.

Additionally, our findings confirmed our hypothesis that antiparkinson medication would significantly increase reach peak velocity, while increasing reach peak velocity. The beneficial medication effects on upper limb velocity are well known, and we replicate this beneficial effect (Baroni et al., 1984; Berardelli et al., 1986; Johnson et al., 1994; Castiello et al., 2000; Kelly et al., 2002; Camarda et al., 2005; Negrotti et al., 2005; Schettino et al., 2006). This finding, in addition to a clear increase in MDS-UPDRS Part III score, made it clear that a 12-h withdrawal period was sufficient for our participants to be in an “off state,” even those on longer-acting medications, like dopamine agonists. Together, these findings suggest that medication has a differential effect on the oculomotor and skeletomotor systems.

### 4.2. Similar effects of bilateral STN-DBS on saccade and reach performance

We found that bilateral STN-DBS improved both saccade performance and one aspect of reach performance (Figure 2). Our findings confirmed our hypotheses that bilateral STN-DBS would significantly decrease saccade latency and increase saccade peak velocity compared to OFF stimulation. Previous studies have also reported that bilateral STN-DBS decreased saccade latency (Fawcett et al., 2007, 2010; Sauleau et al., 2008; Temel et al., 2008, 2009; Yugeta et al., 2010; Antoniades et al., 2012b,a, 2015; Dec-Ćwiek et al., 2017; Goelz et al., 2017; Bakhtiari et al., 2020) and increased saccade peak velocity (Nilsson et al., 2013) in a saccade only task. In addition, bilateral STN-DBS has improved saccadic eye movements during a more complex visual searching task (Tokushige et al., 2018). In the current study, we extend these findings and demonstrate that STN-DBS also has beneficial effects on saccades during a task requiring eye-hand coordination.

Additionally, we confirmed our prediction that STN-DBS would significantly increase reach peak velocity. This is similar to previously reported improvements in upper limb peak velocity (Vaillancourt et al., 2004; Dafotakis et al., 2008; Joundi et al., 2012; Tamás et al., 2016). However, it was surprising that we did not see a larger effect of STN-DBS on reach peak velocity. This was likely because our participants were instructed to move “at a comfortable speed” instead of as fast as possible. This guidance was necessary to prevent participants from hitting the robotic arm that presented the target. Another potential explanation is that the target amplitude was relatively small, only 15° or 11.25 cm away from center, meaning the reach was small. A smaller movement limits how fast a participant can move, even with the beneficial effects of stimulation. Overall, our data suggests that bilateral STN-DBS acts similarly on the oculomotor and skeletomotor systems. The effects of STN-DBS on these systems seem to be beneficial as stimulation brings performance of participants with PD closer to healthy control levels.

### 4.3. The relationship between saccade latency and reach RT

We found a positive relationship between saccade latency and reach RT in the medication group, the STN-DBS group, and in our healthy controls (Figure 3). This relationship was unaffected by medication and STN-DBS. The positive relationship between saccade latency and reach RT has previously been seen in reports on healthy populations (Prablanc et al., 1979; Herman et al., 1981; Biguer et al., 1982; Gielen et al., 1984; Fischer and Rogal, 1986). Specifically, the eyes typically lead the hands (Biguer et al., 1982). In the current study, the initiation of the saccade to the target preceded the initiation of the reach in over 96% of trials for the medication group and over 94% of trials for the STN-DBS group. Therefore, the previously reported positive relationship between saccade latency and reach RT in healthy controls persists in people with PD and remains unaffected by treatment.

The fact that the current study uses a task requiring eye-hand coordination may explain why we found a non-significant increase in reach RT with medication, whereas previous studies have shown a decrease. Simple RT studies have consistently reported that medication significantly (Montgomery and Nuessen, 1990) or non-significantly decreases RT (Velasco and Velasco, 1973; Bloxham et al., 1987; Pullman et al., 1988, 1990; Starkstein et al., 1989; Jahanshahi et al., 1992; Fern-Pollak et al., 2004; Ingram et al., 2021). Even in more complex reaching tasks, in which upper limb RT was measured before a reach to one of multiple targets, medication still decreased RT either significantly (Zappia et al., 1994) or not significantly (Girotti et al., 1986). However, these previous studies did not require explicit eye-hand coordination to complete the task, whereas our task required eye-hand coordination to look and reach to a visual target. As saccade latency was prolonged by medication, it would follow that reach RT would be unable to decrease.

### 4.4. Potential mechanisms underlying the beneficial effects of medication and STN-DBS on upper limb peak velocity

We found that both medication and bilateral STN-DBS improved upper limb peak velocity, as would be expected. The two common pathophysiological models of PD, the rate model and the oscillation model, can both explain this benefit to upper limb peak velocity (David et al., 2020).

Without treatment, PD is characterized by slowness of movement, which is thought to be due to the loss of dopaminergic neurons in the substantia nigra pars compacta projecting to the striatum (Brooks et al., 1990). This results in decreased activity of the basal ganglia direct pathway and increased activity of the indirect pathway (Albin et al., 1989). Combined, the imbalance between the basal ganglia pathways increases the inhibitory activity of the basal ganglia output nuclei, the globus pallidus internus (GPi) and substantia nigra pars reticulata (SNr) (Albin et al., 1989). Therefore, there is excessive inhibition from the basal ganglia output nuclei to downstream targets, such as thalamus, which then results in decreased activation of cortical areas (Albin et al., 1989) and disruption of the motor network (Bologna et al., 2020). It is thought that both antiparkinson medication and STN-DBS reduce the excessive inhibition from the basal ganglia onto the thalamus and reduce the resulting decreased cortical activation, with medication restoring dopamine levels in the striatum and STN-DBS decreasing the overactivity of the indirect pathway. In support of this, positron emission tomography (PET) studies looking at the effects of medication (Jenkins et al., 1992; Rascol et al., 1992, 1994) and STN-DBS (Limousin et al., 1997; Ceballos-Baumann et al., 1999; Grafton et al., 2006) have found that both treatments increase metabolic activity in the motor cortices, especially the supplementary motor cortex. The increased activation of supplementary motor cortex has been correlated with improvement in akinesia with medication (Jenkins et al., 1992) and STN-DBS (Limousin et al., 1997; Ceballos-Baumann et al., 1999), which could explain our beneficial effect on peak velocity.

Additionally, without treatment, PD is also characterized by excessive beta band synchronization throughout the motor loop at rest, both between neurons and in local field potentials (Brown et al., 2001; Levy et al., 2002; Hammond et al., 2007). A proposed explanation of the motor dysfunction in PD is that the excessive tonic beta synchronization prevents the phasic beta suppression that is needed to execute a planned movement (Brittain and Brown, 2014). Medication has been reported to suppress this excessive beta band synchronization at rest (Brown et al., 2001; Levy et al., 2002; Priori et al., 2004; Kühn et al., 2006; Ray et al., 2008), which has been associated with improvement in bradykinesia, akinesia, and rigidity (Kühn et al., 2006; Ray et al., 2008). STN-DBS has also been reported to suppress excessive beta band synchronization at rest in the STN (Eusebio et al., 2011) and throughout the motor loop (Brown et al., 2004; Silberstein et al., 2005). STN-DBS improvement in bradykinesia and rigidity correlates with beta suppression at the cortex during STN-DBS (Silberstein et al., 2005) and following the cessation of STN-DBS (Kühn et al., 2008). The similar mechanistic effects of medication and STN-DBS associated with the beneficial motor effects could also contribute to the reported increase in upper limb peak velocity.

### 4.5. Potential mechanisms underlying the opposing effects of medication and STN-DBS on saccade latency and peak velocity

We found that medication worsened saccade performance by increasing latency and decreasing peak velocity, while STN-DBS improved saccade performance by decreasing latency and increasing peak velocity. In previous studies showing that STN-DBS improves visually-guided saccade performance, the proposed mechanisms are similar to those thought to underlie the improvement in the skeletomotor system after STN-DBS (Sauleau et al., 2008; Temel et al., 2009; Fawcett et al., 2010; Yugeta et al., 2010; Nilsson et al., 2013; Goelz et al., 2017). The worsened visually-guided saccade performance in PD is thought to be due to excessive inhibition on the superior colliculus (Albin et al., 1989; Terao et al., 2011) from the SNr (Hikosaka et al., 2000). STN-DBS reduces the activity of the SNr (Benazzouz et al., 1995; Maltête et al., 2007), which suggests that the superior colliculus would be released from this excessive inhibition. The benefit to saccade performance could also be explained by STN-DBS reducing the excessive beta band oscillations in the basal ganglia, returning the superior colliculus to normal activity levels, and facilitating eye movement (Yugeta et al., 2010). Previous studies have argued that the oscillation model better describes the observed changes in saccade performance in PD and with STN-DBS compared to the rate model (Yugeta et al., 2010). However, as discussed previously, it has been reported that medication also reduces the beta band synchronization in the STN (Brown et al., 2001; Levy et al., 2002; Priori et al., 2004; Kühn et al., 2006; Ray et al., 2008). Therefore, other mechanisms must be contributing to this difference in saccade performance.

It is possible that medication is also acting on other brain areas to counteract the normalization of beta oscillations. As we suggested in a prior publication (Munoz et al., 2022), medication may impair visually-guided saccadic function by overdosing dopaminergic brain regions that are not dopamine depleted in PD. One potential region is the superior colliculus, which has been found to receive dopaminergic projections from the zona incerta and have D2-expressing neurons (Bolton et al., 2015). When dopamine was washed onto the D2-expressing superior colliculus neurons, neuronal activity was suppressed (Bolton et al., 2015). In a different animal model, similar superior colliculus activity suppression resulted in inhibited behavioral responses, such as decreased orientation to stimuli (Glagow and Ewert, 1997). Relatedly, a study examining posterior subthalamic area DBS (PSA DBS) found that, unlike STN-DBS, saccade amplitude and peak velocity were decreased with stimulation (Bangash et al., 2019). It was likely that the zona incerta was being stimulated with PSA DBS (Bangash et al., 2019), which could subsequently inhibit the downstream superior colliculus potentially via an excessive release of dopamine onto the superior colliculus. Taken together, these studies suggest the possibility that the dopamine overdose of the superior colliculus, via medication or stimulation of the zona incerta, could result in worsened saccade performance. Another potential overdosed region is the prefrontal cortex, resulting in increased inhibition from the prefrontal cortex onto the superior colliculus (Hood et al., 2007). Increased dopamine levels in the prefrontal cortex have been shown to result in prolonged saccade latency (Cameron et al., 2018). Additionally, increased inhibition from the frontal cortex has been suggested to inhibit reflexive saccades to allow for sufficient processing time to make a planned saccade (Terao et al., 2013) or to detrimentally increase the focusing of attention, making attention shifting more difficult (Cameron et al., 2018). Conversely, it has been suggested that bilateral STN-DBS may improve visual attention (Tokushige et al., 2018). Dopamine overdose of both the superior colliculus or the frontal cortex could result in an increase in visually-guided saccade latency and a decrease in saccade peak velocity. The overdosing of subcortical and cortical regions is not mutually exclusive and could be occurring simultaneously.

## 5. Conclusion

Using a visually-guided reaching task requiring eye and upper limb movements, we demonstrate that antiparkinson medication adversely impacts saccade performance, while it improves reaching performance. Additionally, we demonstrate that bilateral STN-DBS improves both saccade and reaching performance. Our findings highlight the importance of assessing multiple effectors simultaneously to evaluate how the parkinsonian brain may be affected by treatment. Crucially, the similar and differential effects of antiparkinson medication and STN-DBS on the oculomotor and skeletomotor systems suggest parallel and unique mechanisms of action of antiparkinson medication and STN-DBS. While medication and STN-DBS may impact the basal ganglia circuitry similarly, medication may also overdose other dopaminergic areas resulting in worsened saccade performance.

## Data availability statement

The raw data supporting the conclusions of this article will be made available by the authors, without undue reservation.

## Ethics statement

The studies involving humans were approved by the Northwestern University Institutional Review Board and the Rush University Medical Center Institutional Review Board. The studies were conducted in accordance with the local legislation and institutional requirements. The participants provided their written informed consent to participate in this study.

## Author contributions

MM, LG, DC, and FD contributed to conceptualization and/or methodology of the study. MM, YR, LG, and FD contributed to project administration. GP, LV, SS, and JR provided resources needed for the study. MM, RA, YR, QD, LG, and FD assisted with data collection and/or data processing. MM performed the formal analysis and wrote the original draft of the manuscript. DC and FD supervised the study and the formal analysis. All authors reviewed, edited, and approved the submitted version of the article.
